# Abnormal cortical responses to somatosensory stimulation in medication-overuse headache

**DOI:** 10.1186/1471-2377-10-126

**Published:** 2010-12-30

**Authors:** Gianluca Coppola, Antonio Currà, Cherubino Di Lorenzo, Vincenzo Parisi, Manuela Gorini, Simona Liliana Sava, Jean Schoenen, Francesco Pierelli

**Affiliations:** 1 G.B. Bietti Eye Foundation-IRCCS, Dept of Neurophysiology of Vision and Neurophthalmology, Rome, Italy; 2 Dept of Medical and Surgical Sciences and Biotechnologies, "Sapienza" University of Rome Polo Pontino, Italy; 3 Don Carlo Gnocchi Onlus Foundation, Rome, Italy; 4 Headache Research Unit. University Dept. of Neurology & GIGA-Neurosciences, Liège University, Liège, Belgium; 5 INM Neuromed IRCCS, Pozzilli (IS), Italy

## Abstract

**Background:**

Medication-overuse headache (MOH) is a frequent, disabling disorder. Despite a controversial pathophysiology convincing evidence attributes a pivotal role to central sensitization. Most patients with MOH initially have episodic migraine without aura (MOA) characterized interictally by an absent amplitude decrease in cortical evoked potentials to repetitive stimuli (habituation deficit), despite a normal initial amplitude (lack of sensitization). Whether central sensitization alters this electrophysiological profile is unknown. We therefore sought differences in somatosensory evoked potential (SEP) sensitization and habituation in patients with MOH and episodic MOA.

**Methods:**

We recorded median-nerve SEPs (3 blocks of 100 sweeps) in 29 patients with MOH, 64 with MOA and 42 controls. Episodic migraineurs were studied during and between attacks. We measured N20-P25 amplitudes from 3 blocks of 100 sweeps, and assessed sensitization from block 1 amplitude, and habituation from amplitude changes between the 3 sequential blocks.

**Results:**

In episodic migraineurs, interictal SEP amplitudes were normal in block 1, but thereafter failed to habituate. Ictal SEP amplitudes increased in block 1, then habituated normally. Patients with MOH had larger-amplitude block 1 SEPs than controls, and also lacked SEP habituation. SEP amplitudes were smaller in triptan overusers than in patients overusing nonsteroidal anti-inflammatory drugs (NSAIDs) or both medications combined, lowest in patients with the longest migraine history, and highest in those with the longest-lasting headache chronification.

**Conclusions:**

In patients with MOH, especially those overusing NSAIDs, the somatosensory cortex becomes increasingly sensitized. Sensory sensitization might add to the behavioral sensitization that favors compulsive drug intake, and may reflect drug-induced changes in central serotoninergic transmission.

## Background

Medication-overuse headache (MOH) is a complication of episodic headaches characterized by more than 15 headache days per month and arising from an excessive intake of analgesics or specific anti-migraine drugs, or both [1]. MOH is a disabling health problem that affects 2-4% of the general population and causes considerable long-term morbidity and disability [2]. Most patients attending headache clinics for chronic daily headache have MOH [1,3]. Although MOH evolves from primary as well as secondary headaches the most prevalent initial headache type is episodic migraine without aura and most patients return to the episodic pattern after drug withdrawal [1].

How and why medication overuse leads to chronic episodic headache is unknown. Possible culprits for pain chronification include central sensitization and defective central pain control systems [4]. The addictive behavior and high relapse rates after withdrawal may depend on orbitofrontal cortex hypofunction [5]. The observation that MOH develops predominantly in migraineurs without aura suggests that this headache type possesses pathophysiological peculiarities that could favour drug-induced chronification.

During the pain-free interval in episodic migraine without aura repeated sensory stimuli delivered using various modalities elicit abnormal cortical responses characterized by deficient habituation contrasting with a normal-amplitude initial evoked potential elicited by a small number of stimuli [6]. Current hypotheses attribute this neurophysiological abnormality to cortical hyper-excitability probably arising from deficient intracortical inhibition [7], or to low sensory cortical pre-activation levels ultimately due to abnormal functioning of monoaminergic projections from the brainstem [6,8]. Habituation is considered a protective mechanism intended to prevent neuronal stress and excessive accumulation of metabolites such as lactate and protons that are likely to induce cortical spreading depression or trigeminovascular activation, or both. Evidence suggesting that lack of habituation can promote migraine attacks comes from the observation that it culminates just before the onset of an attack, in the pre-ictal phase [9-11]. During the attack, habituation normalizes, thus transiently activating the protective mechanisms thought to prevent attack recurrence [10-14].

A neurophysiological technique ideally suited to investigate how sensory cortices respond to repetitive stimulation consists of testing somatosensory evoked potentials (SEPs). SEPs are obtained by weak sensory stimuli ideal for disclosing sensitization (reflected by an increased response amplitude to low numbers of stimuli) and habituation (reflected by a decrease in response amplitude after high numbers of stimuli) [15,16], and proved highly sensitive in disclosing abnormal habituation in migraineurs studied interictally, i.e. a clear-cut lack of habituation from the 2^nd ^block of averaged responses onwards [17]. To the best of our knowledge no study has investigated SEP sensitization and habituation in patients with MOH. Having this information may shed light on the mechanisms underlying headache chronification during acute medication overuse.

We used therefore SEPs to investigate whether medication overuse sensitizes the sensory cortices, whether sensitization varies according to the drug overused, and whether the cortical response patterns, sensitization and habituation, differ between patients with episodic migraine without aura recorded in ictal and interictal phases and those with MOH. We also sought possible correlations between the electrophysiological patterns and clinical features including duration of migraine history, duration of headache chronification and class of drugs overused.

## Methods

Subjects-Among consecutive patients attending our headache clinic, 93 patients gave informed consent to participate in the study (Table 1), which was approved by the local ethics committee.

**Table 1:** Demographics data of study participants and headache profiles of patients.

	**HV (n = 42)**	**MOii (n = 41)**	**MOi (n = 23)**	**MOH (n = 29)**	**Triptans (n = 9)**	**NSAIDs (n = 10)**	**Both (n = 10)**
	
Women (n)	26	23	20	23	7	8	8
Age (years)	32 ± 13	34 ± 9	33 ± 12	35 ± 11	32 ± 8	35 ± 9	34 ± 12
Duration of history of migraine (years)		18.0 ± 12.7	16.7 ± 10.9	18.4 ± 11.0	18.3 ± 9.6	22.4 ± 9.2	13.0 ± 13.7
Days with headache/month (n)		2.1 ± 1.9	3.5 ± 2.3	25.9 ± 6.1	22.1 ± 6.2	25.0 ± 7.4	29.4 ± 1.6
Severity of headache attacks (0-10)		6.8 ± 0.8	7.2 ± 1.2	7.2 ± 0.8	7.4 ± 1.1	7.2 ± 0.5	7.1 ± 0.8
Nausea/vomiting (n)		25	16	24	8	9	7
Photophobia (n)		37	21	27	8	10	9
Phonophobia (n)		31	20	27	7	10	10
Pulsating (n)		38	21	26	9	9	8
Duration of the chronic headache (years)				3.0 ± 3.2	1.9 ± 1.8	3.5 ± 3.1	3.3 ± 3.9
Tablet intake/month (n)				74.2 ± 80.8	28.7 ± 16.3	50.5 ± 38.5	127.3 ± 106.5
Motor threshold (mA)	8.4 ± 1.3	8.6 ± 1.3	8.5 ± 1.5	8.7 ± 1.2	8.8 ± 1.3	9.1 ± 1.1	8.3 ± 1.1

Data expressed as mean ± SD. HV healthy volunteers; MOii episodic migraneurs without aura studied interictally; MOi episodic migraneurs without aura studied ictally; N number of subjects

According to the revised ICHD-II criteria [1], 29 patients (35 ± 11 years; 23 women) were diagnosed as having MOH during their first visit, a diagnosis that was confirmed 2 months after withdrawal treatment. These patients were stratified according to the class of drug overused: triptans (n = 9), nonsteroidal antiinflammatory drugs (NSAIDs) (n = 10) or a combination of both (n = 10). Before progressing to MOH, all patients had a clear-cut history of episodic migraine without aura (ICHD-II code 1.1). With the exception of 2 patients who had a mild headache, all MOH patients (n = 27) underwent the SEP recordings in a pain-free state. The 2 patients who had a headache had no associated migrainous features. Because MOH patients tend to take acute medications compulsively and frequently during the day, it was impossible to prevent them from taking a medication on the day of recordings. We managed, however, to perform the recordings at least 3 hours after last medication intake. The 64 patients who had episodic migraine without aura (ICHD-II code 1.1) were assigned to two subgroups: 41 patients (34 ± 9 years; 23 women) were recorded during the interictal period, i.e. at least three days before and after an attack, and 23 patients (33 ± 12 years; 20 women) during the ictal period, i.e. from 12 hours before to 12 hours after an attack. The latter were not allowed to take any acute medication before the end of recordings.

For comparison we recorded SEPs in 42 healthy volunteers of comparable age and sex distribution (mean age: 33 ± 13; 26 women); they had no personal or familial history (1st or 2nd degree relatives) of migraine and no detectable medical condition.

To avoid variability due to hormonal changes, women were recorded outside their pre-menstrual or menstrual periods.

### Data acquisition

SEPs were elicited by electrical stimulation applied to the right median nerve at the wrist using a constant current square wave pulse (0.1 ms width, cathode proximal), a stimulus intensity set at 1.5 times the motor threshold, and a repetition rate of 4.4 Hz. The active electrodes were placed over the contralateral parietal area (C3', 2 cm posterior to C3 in the International 10-20 system) and on the fifth cervical spinous process (Cv5), both referenced to Fz; the ground electrode was on the right arm [18]. SEP signals were amplified with a Digitimer™ D360 pre-amplifier (Digitimer Ltd, UK) (band-pass 0.05-2500 Hz, Gain 1000) and recorded with a CED™ power1401 device (Cambridge Electronic Design Ltd, Cambridge, UK).

Subjects sat relaxed in a comfortable chair in a well-lit room with eyes open. They were asked to fix attention on the stimulus-induced thumb movement. During continuous median-nerve stimulation at the wrist, we collected 300 sweeps of 50 ms, sampled at 5000 Hz. All recordings were averaged off-line using the Signal™ software package version 3.10 (CED Ltd).

Three hundred artefact-free evoked responses recorded in each subject were averaged ("grand average"). After digital filtering of the signal between 0-450 Hz, the various SEP components (N13, N20, P25 and N33) were identified according to their respective latencies. We measured peak-to-peak amplitudes of the cervical N13 component (recorded under the active Cv5 electrode), and the cortical N20-P25 and P25-N33 components (recorded under the active C3' scalp electrode).

**Figure 1:**
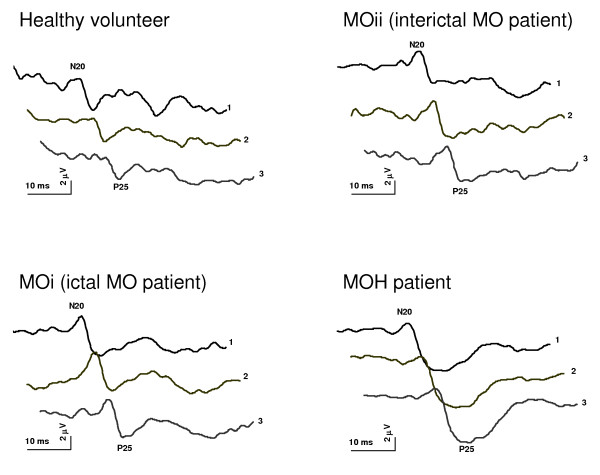
**Illustrative traces of somatosensory evoked potentials habituation in a healthy volunteer, MO Interictally and ictally, and MOH patient**.

Thereafter, the 300 evoked responses were partitioned in 3 sequential blocks of 100 responses (Figure 1). Each block was averaged off-line ("block averages") and analyzed for N20-P25 amplitudes. Sensitization was defined as an increased N20-P25 amplitude recorded during block 1 (after a low number of 100 stimuli), whereas habituation was expressed as the change in N20-P25 amplitude in blocks 2 and 3 compared to block 1 (over a high number of 300 repetitive stimuli).

### Statistical Methods

We used the Statistical Package for the Social Sciences (SPSS) for Windows, version 15.0 for all analyses. For grand average SEPs, component amplitudes were tested in a one-way analysis of variance (ANOVA) with group factor "subjects" (MOH patients, episodic migraineurs without aura studied ictally or interictally, and healthy subjects). To assess changes in SEP amplitude between blocks 1, 2 and 3 SEP N20-P25 amplitudes were tested first with a repeated-measure ANOVA with group factor "subjects" and repeated measures factor "block" then using as group factor "MOH subgroups" (MOH-triptans, MOH-NSAIDs, MOH-combination, and normal subjects). Tukey's test was used for post hoc analyses. Pearson's correlation coefficient was calculated to test correlations between SEP amplitudes or habituation and clinical data (disease duration, days with headache, number of tablets taken per month, duration of chronic headache). P values less than 0.05 were considered to indicate statistical significance.

## Results

**Figure 2:**
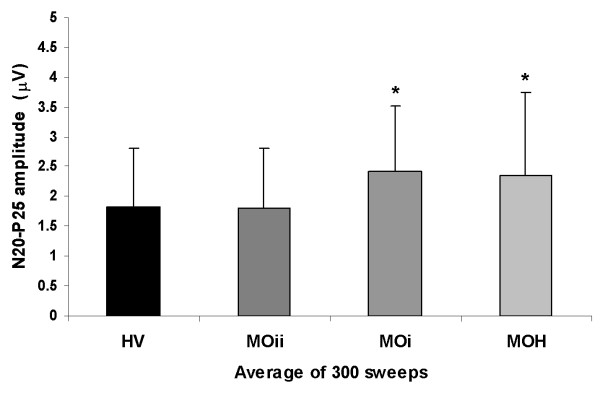
**Somatosensory evoked potential (SEP) amplitude grand average in each study group (HV, healthy volunteers; MOii, migraine without aura interictally; MOi, migraine without aura Ictally; MOH, medication overuse headache; data expressed as mean ± SEM)**.

Assessable SEP recordings were obtained from all patients and controls participating in the study (Figure 1). On grand average SEP recordings after electrical median nerve stimulation latencies of N13, N20, P25 and N33 components were not different between groups (for each measure F(3,131), p > 0.05) whereas their amplitudes significantly differed between groups (F(3,131) = 2.75, p = 0.045). Post hoc analysis showed a higher N20-P25 amplitude in patients with MOH and migraineurs without aura studied ictally than in the subgroup studied interictally and controls (Figure 2).

**Figure 3:**
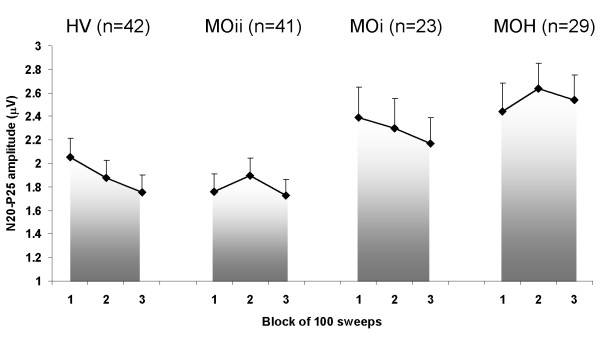
**Somatosensory evoked potential (SEP) amplitude block averages in each study group (HV, healthy volunteers; MOii, migraine without aura interictally; MOi, migraine without aura Ictally; MOH, medication overuse headache; data expressed as mean ± SEM)**.

ANOVA testing SEP amplitude block averages disclosed a main effect for factors group (F(3,131) = 3.83, p = 0.01) and block (F(2,262) = 4.13, p = 0.017), and a significant interaction of group by block (F(6,262) = 2.42, p = 0.027). Post hoc analysis showed in each block a higher N20-P25 amplitude in patients with MOH and migraineurs without aura studied ictally than in the subgroup studied interictally and controls (Figure 3). In controls and migraineurs without aura studied ictally, N20-P25 amplitude decreased from block 1 to block 3, i.e. habituated, while in patients with MOH and migraineurs with aura studied interictally it remained unchanged from block 2 onwards, i.e. did not habituate.

Conversely, ANOVA testing block 1 SEP amplitudes showed a main effect only for factor group (F(3,131) = 2.73, p = 0.046) (Figure 3). Post hoc analysis showed that N20-P25 amplitudes were higher in patients with MOH and migraineurs without aura studied ictally than in the subgroup studied interictally and controls.

**Figure 4:**
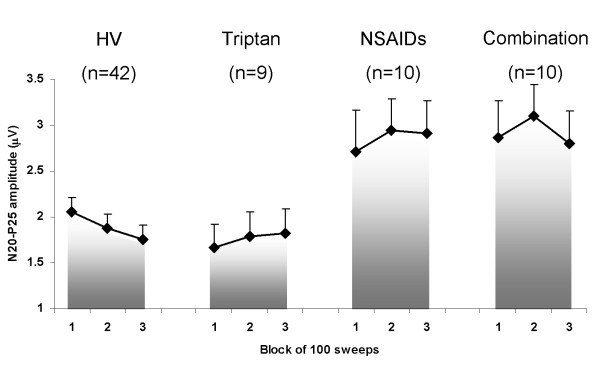
**Somatosensory evoked potential (SEP) amplitude block averages in patients with medication overuse headache (MOH) subgroups and a healthy volunteer (HV) (data expressed as mean ± SEM)**.

When we stratified the data for patients with MOH according to the class of drugs overused, triptans, NSAIDs or both combined, ANOVA for SEP amplitudes in the various blocks, showed a main effect for factor "drug" (F(2,26) = 3.57, p = 0.042). Post hoc analysis disclosed smaller N20-P25 amplitudes in patients overusing triptans than in those overusing NSAIDs or both medications combined. In addition, group analysis between triptan overusers and controls showed that the N20-P25 amplitude in block 1 was normal in patients (F(1,49) = 1.08, p = 0.3) (Figure 4).

Pearson's test disclosed various correlations between SEP amplitude and clinical variables. In patients with MOH, N20-P25 amplitude on SEP grand average correlated negatively with disease duration (i.e. combined duration of episodic and chronic headache phases, r = -0.411, p = 0.046). Conversely, grand average N20-P25 amplitude (r = 0.477, p = 0.016) as well as block 1 N20-P25 amplitude (r = 0.454, p = 0.023) correlated positively with duration of the chronic headache phase.

## Discussion

The distinct changes we found in cortical responses to low and high numbers of sensory stimuli in patients with MOH suggest that the underlying brain mechanisms are altered and differ from those acting in patients with episodic migraine without aura. Low numbers of median nerve electrical stimuli (block 1) disclosed sensory cortex sensitization in patients with MOH and in episodic migraineurs recorded ictally, whereas amplitude changes over sequential block averagings were consistent with habituation in healthy volunteers and episodic migraineurs recorded ictally, but not in MOH patients and episodic migraineurs recorded interictally. In MOH patients, SEP amplitude was lowest in those with the longest history of migraine, whereas it was highest in those with the longest period of headache chronification, suggesting that the electrophysiologic changes reflect chronification. Patients who overused triptans had lower SEP amplitudes than those who overused NSAIDs or both anti-migraine medications combined, indicating that sensitization varies according to the drug overused.

The combination of an initial SEP amplitude increase (sensitization) along with the subsequent lack of habituation suggests that the electrophysiological pattern underlying MOH differs from that underlying episodic migraine. In episodic migraine, SEP recordings show two characteristic changes: a lack of habituation on interictal recordings, and sensitization during the attack. The habituation deficit normalizes during attacks, whereas sensitization disappears between attacks, but in the immediate pre-ictal phase both sensitization and absent habituation may co-exist [9-11]. The electrophysiological pattern we found in MOH may therefore suggest that the sensory cortex is locked in a pre-ictal state associating both hyper-sensitivity (due to sensitization) and hyper-responsiveness (due to deficient habituation), which contrasts with episodic migraine where these cortical states alternate. It is likely that the disclosure of this peculiar electrophysiological pattern was made possible by the fact that we avoided to record MOH patients during a full-blown migraine attack. The SEP pattern associating sensitization and lack of habituation that we compared with a "persistent pre-ictal state", closely resembles the response patterns generated by central sensitized neuronal circuits. Sensitization refers to a facilitatory process that competes with its opposite, habituation to determine the final behavioural outcome after stimulus repetition. This has been called the "dual process" theory [15,16]. Illustrative of central sensitization are the plastic changes in neural structures belonging to the "pain matrix" [19] that result in decreased nociceptive thresholds and increased responsiveness to noxious and innocuous peripheral stimuli [20]. Studies in animals [21] and humans [22] show that SEP amplitudes increase when transient intense activation of nociceptive afferents induces central sensitization, as happens in clinical pain conditions including chronic headache. Our study shows that sensitization, as reflected by increased initial SEP amplitudes, is common to MOH and migraine attacks, although we did not record MOH patients during an attack. A clinical consequence of central sensitization is cutaneous allodynia. It was shown to be prevalent during episodic migraine attacks at cephalic and extracephalic sites [23,24], but even more so in chronic migraine [25]. It is associated with increased nociceptive reflexes [26,27], but, interestingly, in MOH trigeminal evoked potentials were increased, whereas nociceptive blink reflexes remained unchanged, suggesting as in our study that sensitization takes place at supraspinal levels [28].

Our finding that the SEP amplitude increase in MOH is proportional to the duration of headache chronification suggests that medication overuse and increased headache frequency promote or reinforce central sensitization, but leaves open the question of the culprit. Conversely, since total duration of the migraine disorder correlates inversely with SEP amplitudes, the SEP amplitude increase is likely related to factors other than migraine duration and simply repetition of attacks. In keeping with this interpretation, patients who overused triptans alone had no initial SEP amplitude increase indicating that the major culprit for central sensitization in MOH could be NSAIDs. The neurobiological underpinning for this difference remains to be determined. An observation that might favour of NSAIDs consumption as a factor promoting sensitization is that NSAIDs increase spinal expression of inducible cyclo-oxygenase-2 [29], an enzyme that contributes to sensitization in a rat model of inflammatory pain [30].

Another possible link between central sensitization, migraine and anti-migraine drugs is monoaminergic transmission in the central nervous system (CNS). Although both triptan and NSAID overuse lead to headache chronification, only the latter is accompanied by SEP sensitization. We hypothesize that this difference is due to a more profound decrease of 5-HT transmission after NSAID overuse. Between attacks, migraine patients have low blood 5-HT levels whereas the reverse is true ictally [31]. Serotonin synthesis in the brain increases during attacks, and this increase is partly counteracted by acute triptan treatment [32]. Chronic administration of triptans in rats, however, increases 5-HT synthesis in several cortical projection areas of the dorsal raphe nucleus [33] possibly reflecting down-regulation or desensitization of 5-HT1 receptors. By contrast, in rats chronically treated with analgesics, 5-HT2A receptors are down-regulated [34] and the 5-HT transporter is up-regulated in the cortex [34] and in platelets [35]. Upregulated platelet 5-HT transporters [35] and decreased whole blood 5-HT levels [36] tend to normalize after drug withdrawal. Collectively, these experimental data suggest that anti-migraine drug overuse can disrupt central 5-HT transmission. In chronic triptan overuse both pre- and postsynaptic 5-HT1 receptors may become desensitised with the ensuing net effect that serotonergic transmission may be only mildly impaired. During analgesic and NSAID overuse, however, the combination of receptor desensitisation and transporter upregulation may lead to serotonergic hypoactivity. Together with noradrenaline and dopamine, serotonin is crucial for tuning cortical excitability including sensitization and habituation processes and its effect in animals varies with concentration and duration of application [37]. A more severe hypofunction of 5-HT transmission after NSAID overuse may thus explain the SEP sensitisation observed in this subgroup of MOH patients. Whether the difference between the drug classes with regard to central sensitisation is related to the clinical observation that withdrawal headache is much shorter after triptan than after analgesic overuse [38] remains to be determined in a properly designed prospective study comparing clinical outcome and electrophysiological patterns.

The association of electrophysiological sensitisation, i.e. increased 1^st ^block SEP amplitude, and lack of habituation.in MOH patients overusing NSAIDs is intriguing. It is at odds with the electrophysiological pattern associating high amplitude in 1^st ^block and normal habituation found during migraine attacks [10-14], but, as mentioned before, it has been described in the pre-ictal phase [9-11]. One possible explanation for the lack of habituation in episodic migraineurs between attacks is the "ceiling theory" [39] postulating that there is a low preactivation level of sensory cortices, also responsible for the low 1^st ^block amplitudes, would allow a larger range of activation before habituation occurs [6,8]. The habituation deficit in NSAIDs overusers cannot be explained by the "ceiling theory" since their high 1^st ^block amplitude indicates rather that the somatosensory cortex is sensitised. There is at present no straight forward explanation for this pattern. It is likely, however, that other neurobiological mechanisms that participate in the production of habituation are impaired. For instance, inhibitory interneurons could be hypofunctioning because of the reduction in serotonergic transmission induced by the prolonged NSAID overconsumption. This hypothesis can be tested experimentally by searching if habituation normalizes during full-blown attacks in MOH patients like in episodic migraine and by exploring inhibitory cortical interneurons with dedicated neurophysiological studies such as that of cortical silent periods using transcranial magnetic stimulation. Given the similar neural mechanisms underlying sensory and behavioural sensitization [40], the interesting question arises whether the sensory sensitization in patients with MOH parallels behavioural sensitization. Behavioural sensitization is paradigmatic of how the serotonergic, dopaminergic, and noradrenergic systems interact and contribute to central sensitization [41]. Brain circuits involved in addictive behaviour include ventral and dorsal striatum, amygdala and orbitofrontal cortex and are heavily modulated by dopaminergic projections from the ventral tegmental area of the midbrain, serotonergic projections from the median and dorsal raphe nuclei, and noradrenergic projections from the locus coeruleus [4,42]. According to DSM-IV criteria, many MOH patients manifest a dependence behaviour [43]. The latter has been associated with orbito-frontal cortex hypoactivity [44], an abnormality also found in subgroups of MOH patients [5]. The orbito-frontal cortex is thought to modulate habituation mechanisms [45] and orbito-frontal lesions induce SEP sensitization and lack of habituation [46], precisely the two sensory abnormalities we found in patients with MOH. Our findings along with current knowledge on the neurobiology of drug overuse therefore suggest that future studies seeking correlations between electrophysiological and metabolic measures should focus on the orbito-frontal cortex. In our study we did not control for associated depression and anxiety. Despite the evidence that cortical pain-related evoked potentials in MOH do not differ between subgroups of patients with or without depressive symptoms [28], it may still be appropriate to control for psychiatric comorbidity in future studies.

## Conclusions

Cortical responses to repetitive sensory stimuli are abnormal in patients with MOH. Increased response amplitudes after low numbers of stimuli indicate sensory sensitization and lack of amplitude decrease during subsequent stimulations reflects a habituation deficit. This cortical response pattern is similar the one found in the immediate pre-ictal phase in episodic migraine, but different from the interictal and ictal patterns. It suggests that the somatosensory cortex has become persistently sensitized and that the migraine generating mechanisms in the central nervous system are not shut off. The sensitization is obvious in patients overusing NSAIDs and almost non-existent or masked in those who overuse only triptans. The different electrophysiological pattern between drug classes may be related to the clinical observation that withdrawal headache is shorter lasting in triptan overusers than in NSAID overusers. We postulate that the abnormal sensory processing in MOH patients reflects a drug-induced impairment of central serotonin neurotransmission, that the decrease of serotonergic activity is more profound after chronic NSAID overconsumption and that the cortical sensory sensitization parallels the behavioural sensitization that accompanies drug overuse and is crucially modulated by the medial orbitofrontal cortex.

## Competing interests

The authors declare that they have no competing interests.

## Authors' contributions

GC made substantial contributions to acquisition of data, analysis and interpretation of data as well as in drafting the manuscript. AC, VP, JS and FP were implied in the interpretation of data as well as in drafting the manuscript; gave critical revision of the manuscript for important intellectual content. CDL, MG and SLS were implied in recording data. All authors read and approved the final manuscript.

## Pre-publication history

The pre-publication history for this paper can be accessed here:

http://www.biomedcentral.com/1471-2377/10/126/prepub
